# Extracellular traps and the role in thrombosis

**DOI:** 10.3389/fcvm.2022.951670

**Published:** 2022-08-25

**Authors:** Tonglei Han, Hanfei Tang, Changpo Lin, Yang Shen, Dong Yan, Xiao Tang, Daqiao Guo

**Affiliations:** Department of Vascular Surgery, Zhongshan Hospital, Fudan University, Shanghai, China

**Keywords:** extracellular traps, neutrophil, macrophage, thrombosis, venous

## Abstract

Thrombotic complications pose serious health risks worldwide. A significant change in our understanding of the pathophysiology of thrombosis has occurred since the discovery of extracellular traps (ETs) and their prothrombotic properties. As a result of immune cells decondensing chromatin into extracellular fibers, ETs promote thrombus formation by acting as a scaffold that activates platelets and coagulates them. The involvement of ETs in thrombosis has been reported in various thrombotic conditions including deep vein thrombosis (DVT), pulmonary emboli, acute myocardial infarction, aucte ischemic stroke, and abdominal aortic aneurysms. This review summarizes the existing evidence of ETs in human and animal model thrombi. The authors described studies showing the existence of ETs in venous or arterial thrombi. In addition, we studied potential novel therapeutic opportunities related to the resolution or prevention of thrombosis by targeting ETs.

## Introduction

Thrombosis is a major contributor to the global disease burden because one in four people die from thrombotic conditions (1). As a result of blood clots impeding normal blood flow in the arteries or veins, thrombosis causes conditions such as ischemic stroke, ischemic heart disease, and venous thromboembolism (VTE). Previously, thrombosis was only considered as a vessel or blood disease. The discovery of extracellular traps (ETs) has significantly changed our understanding of thrombosis. ETs, net-like structures formed from DNA and proteins, studded with histones and cellular proteins, are released by many types of immune cells (2–4). Up to now, much more immune cells have been confirmed to have the ability to form ETs, including mast cells (5), eosinophils (6), monocytes (7) and macrophages (8) etc. After activating stimuli including exogenous microorganisms (bacteria, virus, fungi, and parasites), the immune complexes are released, these cells will undergo a new type of programmed cell death “ETosis” and then release ETs (2, 9–11).

The first evidence for neutrophil extracellular traps (NETs) appeared in 2004 (12). ETs ensnare both gram-positive and gram-negative bacteria, and myeloperoxidase (MPO) or neutrophil elastase (NE) degrades bacterial virulence factors (12). ETs, recently considered as a double-edged sword, were initially found to be capable of immobilizing and killing microorganisms and are also involved in the pathology of many diseases, such as autoimmune diseases (13), occlusions (14), aseptic inflammation (15), and even in coronavirus disease 19 (COVID-19) thrombosis (16). Growing evidence indicated that ETs were present in thrombi from animal models or in patients with thrombosis and played an important role in thrombotic diseases (17–20). There are three forms of ETs: aggregated ETs (aggETs), full-size ETs, and ET remnants. However, the main prothrombotic inducer remains debatable (21). Nevertheless, many important functions of these different types of ETs, such as NETs, macrophage/monocyte extracellular traps (METs), and mast cell extracellular traps (MCETs), remain incompletely understood. For better prevention, diagnosis, and treatment of thrombosis, a deeper understanding of its underlying mechanisms is crucial. This article aimed to describe the ETs formation process, review the roles of ETs in thrombosis, and identify possible drugs that could antagonize ETs.

### What is ETosis and ETosis stimuli

**FIGURE 1:**
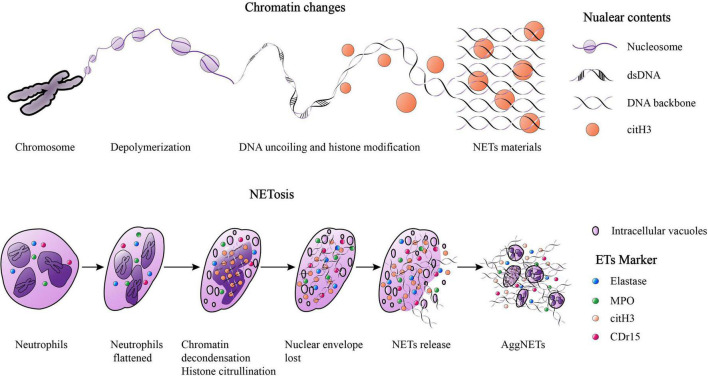
The Chromatin changes when NETosis and the process of NETosis. When “NETosis” occur, the chromosomes in nuclear will experience the following phases: chromatin depolymerization, DNA uncoiling and histone modification, finally forming net-like structures. The NETosis process include: isolated neutrophils flattened, the formation of intracellular vacuoles, chromatin decondensation, histone citrullination, the loss of the nuclear envelope, the mixing of cytoplasmic granular proteins and nuclear contents, membrane integrity destroyed, and ultimately, NETs were released. A large amount of NETs gathered and formed the “aggNETs.”

The first evidence for the ETosis mechanism of neutrophils was reported in 1996, in which cell death of neutrophils could be induced by phorbol myristate acetate (PMA) for as little as 10 min (22). However, later studies have suggested that ETosis could take several hours (9, 12). The ETosis process includes flattening of isolated neutrophils, formation of intracellular vacuoles, chromatin decondensation, histone citrullination, loss of the nuclear envelope, cytoplasmic granular proteins mixed with nuclear contents, destruction of membrane integrity, and finally release of ETs (23). “NETs” are used as an example here (Figure 1). It’s worth noting that a study reported that not all ETosis pathways ended in cell death (24). Some evidence also suggests that the formation of NETs was not a direct cause of cell death (25, 26). Thereby, ETosis was considered as a new active process which different from apoptosis or necrosis and resulted in substantial morphological changes including signs of chromatin decondensation, mixing of nuclear, granular, and cytoplasmic components (9, 22).

Even numerous triggers were reported, the pathways of the formation of NETs are still under debate and the key molecules essential are also difficult to clearly explain the specific mechanism of NETosis. In addition to PMA, interleukin (IL)-8, and lipopolysaccharide (LPS) were also found to be capable of activating NETosis, and extracellular structures identified as fragile fibers of decondensed DNA decorated with granule proteins and histones could be observed (12, 27, 28). IL-1 receptor antagonist significant decreased NETs release from neutrophils *in vitro* and the recombinant IL-1 treatment induced NETs formation again (29). IL-6, was indicated to be an inducer of the formation of energy dependent NETs (30). IFN-r, was verified to be a NETs formation modulator (31). Fewer NETs were observed in the bronchoalveolar lavage fluid of IFN-r^–/–^ mice vs. wild-type mice (32). Neutrophils were still viable when the nuclear lobular shape was lost and chromatin expanded in the cytoplasm; before rupture of the plasma membrane, calcein blue was still present but negative for annexin V (9). Reactive oxygen species (ROS) are reported to be an important prerequisite for NETs formation (33). NETs cannot be produced by neutrophils with mutations in the NADPH oxidase of phagocytes when stimulated by PMA, but can be restored after hydrogen peroxide is added. This study indicated that the activation of NADPH oxidase is essential for NETosis (33). Hakkim et al. reported that protein kinase C (PKC) signaling via the RafMEK-ERK pathway can also activate NETosis (33). Both ROS and MPO are essential for NE activation (34). NE can degrade core histones and facilitate the decondensation of chromatin and citrullination of histones in synergy with the calcium-dependent enzyme peptidyl arginine deiminase 4 (PAD-4) (35). These results indicate that ROS, MPO, NE, and PAD-4 play important roles in NETosis. A previous study showed that PAD-4 activity is necessary for NETs formation (36). After PAD-4 is knocked out or inhibited, the formation of human or mouse NETs is disrupted (37–39). Notably, excessive NETs were observed in influenza A and COVID-19 virus-induced lung inflammation (40–43). This indicated virus might be a stimulus for NETs formation. However, several studies have observed that under the same stimulus, the formation can be independent of the activity of PAD-4 (44, 45).

### Neutrophils formed neutrophil extracellular traps in acute inflammation

Neutrophils are the first responders and critical fighters of the innate immune system, and are heterogeneous and abundant leukocytes that participate in acute inflammation, including infection and injury (46). Approximately 60% of leukocytes are circulating neutrophils, released from the bone marrow and with a short lifespan (47, 48).

In 2004, a novel substance NETs produced by neutrophils that underwent NETosis was discovered *in vitro* (12). NETs are composed of filamentous DNA scaffolds, granular proteins and histones (12). Many substances have been explored to determine whether they can promote or inhibit NET formation. PMA has been verified many times and has been used to induce NETs formation (49). Andzinski et al. reported that type I interferons (IFNs) display a proinflammatory subset of neutrophils and enhance NETs formation (50). Boufenzer et al. (23) demonstrated that the activation of TREM-1 increases the formation and release of NETs, promotes vascular dysfunction, and activates endothelial cells. The direct interaction between platelets and neutrophils during platelet-driven NETosis is mediated mainly by P-selectin/PSGL-1 and GPIbα/Mac-1. Soluble mediators released by platelets, including high mobility group box 1 (HMGB1), regulated on activation normal T-cell expressed and secreted (RANTES) and platelet factor 4 (PF4), further stimulated NETs formation (51–53). Previous studies demonstrated that platelets had the ability to directly stimulate the production of NETs (52, 54). Following the above findings, platelet-derived exosomes in patients with sepsis were shown to promote excessive formation of NETs and subsequently result in organ injury (55). Kono et al. (56) reported that deferasirox (DFS) is an iron chelator that can inhibit ROS production and NETs formation. Several studies on the intracellular regulatory mechanism of NETs formation demonstrated that a lack of miR-146a targeting the TLR4 signaling pathway in mice could effectively prevent the formation of NETs (57, 58). As a result, platelet TLR4 detected TLR4 ligands in the blood and activated neutrophils, forming NETs (59). Although NETs have received increasing attention, most relevant studies have only focused on animal and *in vitro* experiments (60). The clinical value of NETs in thrombosis requires further exploration.

### Macrophages, monocytes and mast cells can also form extracellular traps

The tissue macrophage differs from the circulating blood monocyte, which is derived from bone marrow or originates from tissue-based embryonic precursors and is maintained independently of bone marrow progenitor cells (61–63). Macrophages are involved in many functions, such as maintaining homeostasis, tissue repair, and immune regulation (64–66).

Current studies have reported that macrophages that undergo METosis could also produce ETs, which were named METs (2). METosis occur rapidly within 30 min (67, 68). The METs are produced by macrophages when they encounter microorganisms and are similar to NETs, which are also composed of DNA met-like structures. METs can immobilize and kill microorganisms, but may also contribute to disease pathology. In 2008, a study first reported that mast cells *in vitro* could produce NETs like extracellular structures which were named MCETs with antimicrobial activity (3). The formation of MCETs in mast cells was later reported when exposed to other GAS strains (69) or to other extracellular bacteria. For instance, BMMCs or HMC-1 infected with *S. aureus* (70), HMC-1 in contact with Pseudomonas aeruginosa (3), or BMMCs co-cultured with *Enterococcus faecalis* (71). The bacteria trapped in MCETs are killed (70, 71). Nakazawa et al. (72) demonstrated that macrophage polarization may affect METosis. The M1 activated state is more prone to METosis when exposed to NETs materials.

With the exception of live bacterial cells, Wong et al. (73) reported that METs were released from bacteria when they were exposed to specific virulence factors, such as ESX-1 and the secretion system of Mycobacterium tuberculosis. It was also demonstrated that mouse RAW264.7 and J774A.1 macrophage-like cells incubated at 42°C for 1.5 h and followed by recovery at 37°C were more prone to form METs co-cultivated with Streptococcus agalactiae (57). Halder et al. (67) reported that METs released from monocytes have an important host defense function which is capable of inhibiting fungal growth. Previous studies have shown that METs can be formed if proinflammatory mediators induce the production of ROS, although these findings were not consistent across all experimental conditions and cell types (74, 75). However, why some macrophages develop METosis while others do not remains controversial. To date, an increasing number of types of immune cells have been demonstrated to have the ability to form ETs; however, the specific mechanisms and functions of different types of ETs remain unclear.

### Specific markers for identifying extracellular traps and several methods for detecting extracellular traps formation

To clarify the distribution of ETs in tissues and detect ETs content, it is necessary to search for specific markers for subsequent research. Several known ET components, including citrullinated histones, elastase, or MPO, are stained in the additional verification proposal. Although the circulating concentrations of these markers were easily influenced, they were still the most commonly used in many studies (11, 76). Citrullinated histone H3 (citH3) has been identified as one of the most specific markers for ETs (77–79). Elastase was originally considered a neutrophil-specific marker, but has also been discovered in human macrophage-like cells and peripheral blood monocytes (67). In addition, MPO is also found in METs produced from a variety of macrophage populations, including glomerular macrophages, monocytes, J774A.1 macrophage-like cells, and caprine monocytes (67, 68, 80, 81). A recent study reported that CDr15 in the plasma could be a new marker for detecting NETs (82). The surrogate markers for ETs are plasma cell-free DNA, including double-stranded DNA (dsDNA), histone-DNA complexes, and chromatin, which have often been estimated in clinical trials in recent years (83–86). Recently, there were also studies reported that MPO-DNA complexes and cit-H3-DNA complexes in serum samples could be treated as a useful biomarker of serum NETs level (87).

ELISA is the screening test that is commonly used for detection of serum markers. However, there has been a study indicating that ELISAs for MPO, NE and nucleosomes do not specifically determine the formation of NETs, and the standardization of tests for MPO-DNA and citH3 is problematic (88). Recently, flow cytometry analysis has gained increasing interest for NETs identification and quantification. There is no potential for observer bias in this new methodology since it allows rapid and robust assessment of several thousand cells per sample, and it is independent from potential observer bias. To assess the formation of NETs *in vivo*, detecting NETs components on extracellular vesicles (EVs) (89) and cells (90, 91) by using flow cytometric assays may be an alternative method. Otherwise, for assessing and visualizing the formation of NETs, live cell imaging, conventional microscopy, intravital microscopy, and scanning or transmission electron microscopy are commonly used imaging-based applications (9, 88, 92). The drawback of such approaches is that they are difficult to precisely distinguish if DNA-protein complex from NETs other than another kind of cell death (93–95).

### Extracellular traps in venous thromboembolism

VTE, which mainly includes deep vein thrombosis (DVT) and pulmonary embolism (PE) (96), ranks third among all causes of acute cardiovascular syndrome (97). DVT causes post-thrombotic syndrome, whereas PE causes chronic pulmonary hypertension. Both these conditions affect quality of life. Recently, the role of ETs in VTE has gained increasing attention, and the following describes DVT and PE.

#### Extracellular traps are abundance present in deep vein thrombosis

DVT is a prevalent disease worldwide with severe major complications accompanied by high morbidity and mortality. The Virchow’s triad, including hypercoagulability, vascular dysfunction, and stasis, is considered an excellent guide to understand thrombotic risk factors (98). Recently, dysregulation of the immune system was suggested to be considered in the process of thrombosis (99).

In human venous thrombosis, NETs act as fibrous scaffolds for von Willebrand factor (vWF), fibrin, and platelets. This is the first report to confirm that NETs have a prothrombotic (11). A case report first confirmed that NETs were abundant in human thrombus samples, especially in patients with DVT and microscopic polyangiitis (100). Savchenko et al. (96) collected 16 thrombi obtained from 11 patients during autopsy or surgery. Histological analysis showed that a large number of DNA web structures and citH3 were concentrated in the thrombi organizing parts, with only a few in already organized sections. These results indicate that thrombi development is associated with NETs. In some animal experiments, researchers have obtained similar findings. Brill et al. (101) collected samples from patients with chronic thromboembolic pulmonary hypertension patients and a vena cava ligation mouse model. The NET-specific marker citH3 was abundant and co-localized with vWF in thrombi, especially in the fresh parts. Similarly, large amounts of NET-like structures were observed in thrombi in a mouse DVT model (102). Therefore, dissolution of NETs may facilitate thrombolysis. Based on these findings, plasma DNA levels are clinically used in the diagnosis of DVT with a rather high sensitivity of 81%, and the DNA in thrombi is positively correlated with vWF activity, D-dimer level, neutrophil activation, and clinical Wells score (103, 104).

#### Extracellular traps have prothrombotic effect in pulmonary embolism

PE is a severe complication of DVT (105). The level of nuclear DNA in circulation is significantly elevated in patients with PE, and there is a clear positive correlation with mortality (106, 107). In patients with chronic thromboembolic pulmonary hypertension, neutrophils were found to exist on the surface of thrombi with high reactivity, and soluble NETs surrogates were significantly increased compared to healthy controls (108). Furthermore, a plasma test in patients with diabetes mellitus verified that NETs were mainly formed in the early stages of thrombosis, especially in the acute stage, which was different from ETs produced by other immune cells (109). Using a murine orthotopic 4T1 breast cancer model, Cao et al. (110) demonstrated that dunnione (a strong substrate of NADPH quinone oxidoreductase 1) could inhibit NETs formation and the subsequent occurrence of PE by decreasing cellular NAD levels. These findings confirm that ETs are involved in PE, especially in the progression of thrombosis. Additionally, controversial studies have suggested that ETs may be associated with outcomes in patients with PE. A clinical study that enrolled 25 healthy controls and 126 normotensive acute PE patients revealed that high circulating citH3 levels enhanced NETs formation and were associated with an increased risk of acute PE-related death (111). In recent years, the coronavirus disease (COVID-19) epidemic has spread globally. Several researchers found that thrombotic complications are associated with severe COVID-19 morbidity and mortality (112, 113). However, Prevel et al. (114) reported that in 50 hospitalized COVID-19-related ARDS patients, plasma markers such as cell-free DNA, MPO–DNA complexes, and citH3 were associated with survival but not with PE. As there is a lack of research on ETs in COVID-19 and limited by sample size, further in-depth studies are needed. Above all, these findings prove that ETs contribute to the progression of thrombosis and may be potential therapeutic targets for preventing PE.

### Extracellular traps in arterial thrombosis

Clinical challenges remain in the prevention and treatment of arterial thrombosis. Understanding the relevant molecular mechanisms can help identify new targets and therapeutic approaches that can improve protection against severe thrombotic events. The role of ETs has mostly been studied in the following common arterial thrombosis diseases: acute myocardial infarction (MI), acute ischemic stroke, abdominal aortic aneurysm (AAA), peripheral artery disease (PAD), and thrombotic microangiopathies (TMAs).

#### Extracellular traps contribute to the pathological progression of acute myocardial infarction

Abundant neutrophils and NETs have been discovered in fresh culprit site thrombi in patients who have died from MI (115–117). NETs combined with pro-inflammatory IL-17 drive the accumulation of neutrophils (118) and have been suggested to play an important role in the pathogenesis of MI (119). A substantial burden of NETs has also been observed in thrombectomy samples from patients with stent thrombosis after percutaneous coronary intervention (120). Several clinical studies have indicated that NETs can promote thrombosis of the coronary microvasculature and damage heart function (121–123). Abo-Aly et al. (124) enrolled 22 ST-segment elevation MI patients who underwent percutaneous coronary intervention in an open-label prospective randomized study. Patients’ total elastase, MPO-elastase complexes in plasma, and thrombolysis in myocardial infarction flow scores were demonstrated to be significantly reduced after receiving a dose of 30 mcg/kg bolus cangrelor followed by a 4 mcg/kg/min intravenous infusion from the start of intervention to 2 hours later (124). Previous studies have revealed the presence of ETs in the thrombus and plasma of patients with MI, and found possible relationships between ETs and adverse events. Whether monitoring of these ETs markers could help prevent adverse events need further exploration.

In addition, if ETs play an important role in the pathological progression of MI, it might be a selective therapeutic target for developing new drugs to prevent MI-relevant thrombosis events. However, whether ETs are simply a marker of adverse prognosis is still unclear (125). A clinical study confirmed that NETs were present at the culprit site of acute MI thrombus, and the formation of NETs was induced by HMGB1, which is an important risk-related molecular pattern (126). Compared with venous thrombi, coronary artery thrombi had a significantly higher NETs burden and were positively correlated with infarct size (127), as well as in patients infected with COVID-9 (128). Recently, the dsDNA level measured 1 d after MI was also found to be related to the myocardial salvage index, left ventricular ejection fraction, and microvascular obstruction during follow-up (129). *In vitro*, neutrophils collected from the culprit lesion site showed a greater tendency to develop NETosis compared to neutrophils from non-infarcted coronary arteries (130). By activating and differentiating fibrocytes, NETs also contribute to myocardial fibrosis at the culprit site (77). Notably, several previous studies had already confirmed that neutrophil:lymphocyte ratios, peripheral neutrophil count, and NETs-related markers correlated with adverse cardiovascular outcome (131, 132). And meanwhile, there are also evidence demonstrate that markers of ETs, neutrophil activation and DNase activity are related to inflammatory indicators, such as interleukin-6 and C-reactive protein (133). Above all, MI is a complex progression involving numerous inflammatory factors, if ETs played a major role is still debatable. Otherwise, a considerable proportion of patients with MI have a history of chronic coronary heart disease (CHD). Many studies have focused on NETs in the acute stage, and there is still a lack of research on the role of other types of ETs in the long-term progression of MI.

#### Extracellular traps-induced thrombosis may be the cause of acute ischemic stroke

Strokes caused by thromboembolic occlusions of the middle cerebral artery (MCA) and/or internal carotid artery (ICA) are the second leading cause of death and morbidity worldwide (134). Abundant NETs were discovered in thrombus samples taken from patients with ischemic stroke, and a large number of neutrophils were positive for citH3 in these thrombectomy samples (135, 136). Deng et al. (137) also verified that NETs were present in thrombi obtained from patients with acute ischemic stroke. Inhibition of NETs formation could decrease infarct size in a mouse permanent middle cerebral artery model. There is increasing evidence that NETs can antagonize tissue plasminogen activators by stabilizing clot-induced thrombus formation and promoting coagulation, a frequently encountered problem in the treatment of stroke patients (138–140). This demonstrates that NETs are involved in the pathogenesis of cerebral occlusion. Otherwise, a high level of plasma DNA indicated that patients were at risk of death at follow-up (141, 142), whereas nucleosomes were related to infarction volume and neurological dysfunction (143). Tsai et al. performed a clinical control trial that included 50 patients with acute ischemic stroke and 50 at-risk controls. The results demonstrated that plasma DNA significantly increases after stroke (144). Vallés et al. (145) conducted a clinical study of 243 patients with acute ischemic stroke. They determined NETs markers, including citH3, nucleosomes, and cell-free DNA in plasma. The 12 months follow-up results showed that patients with high citH3 levels had an increased all-cause mortality. Prognosis and outcome were associated with soluble NETs markers, which are indicators of stroke severity based on the National Institutes of Health Stroke Scale (NIHSS). These findings show that close monitoring of plasma ETs markers is essential, and effective treatment of ETs might prevent the occurrence of acute ischemic stroke.

#### Extracellular trap is a key factor in thrombosis in abdominal aortic aneurysm

AAA, an arterial disease related to thrombosis with potentially fatal consequences of aortic rupture, is characterized by multilayer intraluminal thrombosis and vessel dilation (146). An intraluminal thrombus, whose growth is accelerated by turbulent blood flow, damages aneurysmal walls by immune-inflammatory pathways (147). In human AAA, Delbosc et al. (148) revealed that neutrophil activation resulted in NETs formation in the intraluminal thrombus, leading to cell-free DNA release. Consistent results were obtained in the rat AAA model. Meher et al. (149) performed further clinical research on human AAA thrombi. NETs were found to co-localize with IL-1β, a pro-inflammatory mediator that drives the process of AAA and accelerates the formation of NETs. Johnston et al. (150) verified a similar result and explored the mechanism in an elastase perfusion mouse AAA model. This proved that genetic deletion or receptor antagonism of IL-1β reduced NETs formation and AAA initiation and progression. Fernández-Ruiz (151) also confirmed that NETs stimulated by IL-1β aggravate the formation of AAA in a mouse AAA model induced by elastase perfusion. Despite the association between AAA and thrombotic disease, most clinicians and researchers focus only on the etiological mechanisms of arterial dilation. Other pathological evidence related to NETs in aneurysm thrombosis is scarce and requires further investigation.

#### Extracellular traps aggravate peripheral artery disease and thrombotic microangiopathies

In general, PAD refers to diseases of the non-coronary vasculature which can be aneurysmal, atherosclerotic, inflammatory, or a combination of pathologies (152). Farkas et al. (153) demonstrated that NETs levels in PAD thrombi were similar to those in the coronary arteries and stroke thrombi. On comparing plasma samples from patients with PAD and DVT, neutrophil elastase alpha1 anti-trypsin complex, showed a significant increase in PAD (154).

TMAs are a group of rare, but serious disorders can be caused by a variety of clinical events, such as disseminated intravascular coagulation, hemolytic uremic syndrome, etc. (155). Fuchs et al. (156) conducted a cohort study of plasma samples from 10 healthy controls and 29 acute TMAs patients. Research has shown that increased plasma extracellular DNA and histones are associated with the activity. Thrombosis in COVID-19 patients affected both arterial and venous circulation, resulting in stroke, acute coronary syndrome, DVT, PE, and TMAs (157, 158). A prospective cohort study compared plasma citH3, cell-free DNA, and MPO-DNA complexes in patients with healthy controls (43). The authors indicated that MPO-DNA complexes increased in COVID-19 and correlated directly with illness severity. NETs have prothrombotic clinical presentations and aggregate occluded small pulmonary vessels widely in the lungs of patients with COVID-19-related acute respiratory distress syndrome (43). To date, there is still a lack of research on ETs in PAD and TMAs; however, it has been verified that ETs are involved in the progression of such thrombotic diseases.

## Extracellular traps as a potential therapeutic target

It is essential to find therapeutic compounds that inhibit ETs and block their detrimental effects since ETs play such a crucial role in thrombotic diseases. There have been several pathways proposed to target different ET components. Therefore, the mechanism of ETs formation must be explored for every single condition, and specific therapeutic strategies must be developed for every single condition. Except for DNA bone structure, the histones derived from NETs should also be noted. Previous studies demonstrated that histones within NETs played a cytotoxic role in the pathogenesis of endothelium damage (59, 159). Excessive NETs formation can be suppressed by treating NE, MPO, PAD4, and extracellular DNA (160, 161). Developing drugs, inhibiting PAD4, and treating with Deoxyribonuclease (DNase) 1 are the most promising treatments for NETs-induced occlusions (162).

### The inhibition of PAD4 decrease the formation of extracellular traps

A family of enzymes called peptidylarginine deiminases (PADIs) is involved in post-transcriptional deamination/citrullination. A positively charged arginine molecule is converted to an uncharged citrulline molecule in this process. Among the five members of PADI, PAD4 is unique. By decondensing chromatin, it contributes to NET formation, as evidenced in the *in vitro* studies of peripheral blood neutrophils (163). This makes PAD4 an attractive therapeutic target for treating occlusive NET-related diseases. A synthetic inhibitor of PAD4 called Cl-amidine has been used in the treatment of rheumatoid arthritis in mammals for decades (164, 165). Cl-amidine effectively blocked the synthesis of NETs based on its irreversible blocking properties in both *in vitro* neutrophils and murine lupus models *in vivo* (163, 166, 167), and disease severity was effectively attenuated in mouse models of various diseases, such as cardiovascular events (166), sepsis (168), and collagen-induced arthritis (169). Administration of Cl-amidine prevented thrombotic occlusion in a mouse model of ischemic stroke by promoting NET lysis (170). Although conflicting results exist, there is a certain dependence of NETosis on enzymatic PAD4 activity, making PAD4 inhibition a potentially promising treatment for human thrombotic disease.

### Deoxyribonuclease can degrade extracellular traps

The Deoxyribonuclease (DNase) family, including DNase1 and DNase1-like 3 (DNase1L3), is a class of enzymes that can degrade ETs (170). Non-hematopoietic tissues express DNase1 and it preferentially degrades protein-free DNA (4, 171). Immune cells secrete DNase1L3, also called DNase gamma, which cleaves protein-DNA complexes (4, 172). DNase degrades NETs by hydrolyzing the DNA backbone (11) and reduces the size and amount of aggNETs (173). Lack of dual host protector DNases *in vivo* cannot remove ETs efficiently, and aggETs can occlude vessels (21). The homeostatic balance between NET formation and degradation is dependent on adequate DNase activity (174). The mice were protected from vascular occlusion in either reconstitution with or the presence of only one functional type of DNase (170). It has been demonstrated that targeting chromatin and NETs with DNase 1 in several mouse or rat disease models is beneficial for experimental DVT (101, 102) and ischemic injury, including testicular torsion (175) and ischemic stroke (174). Shrestha et al. (176) demonstrated that NETs exert antitumor effects in a murine tumor model. Retinoic acid can induce hypersegmented neutrophils which enhances NETs formation and cytotoxicity against tumor cells in a murine tumor model. However, the addition of DNase reversed this antitumor effect because of NET degradation. The results of this study demonstrated that DNase could effectively remove NETs and that the balance between DNase and NETs plays an important role in cancer diseases. Ge et al. (177) reported that the use of DNase 1 alone could reduce neutrophil infiltration in a rat model of ischemia/reperfusion, and that cardiac function was improved by the co-administration of tissue plasminogen activator (tPA). In contrast, another study indicated that in mice with coronary artery ligations, DNase 1 treatment improved cardiac function without reducing neutrophil infiltration into ischemic myocardium (178). It is possible that these apparently contradictory results are due to variations in the ligation methodology, the duration of ischemia, and the timing of the DNase treatment. *In vitro*, DNase 1 accelerated thrombolysis mediated by tPA in human coronary (127) and cerebral (135) thrombi compared with tPA alone. Furthermore, patients with MI experiencing low DNase activity typically have a larger infarct size (127). Taking into account the growing evidence for the benefits of applying DNase in well-established models of disease. Patients with neutrophil-driven disease, in which NETs play a pathogenic role, may benefit from DNase 1 treatment.

### Heparin has the effect of antagonizing neutrophil extracellular traps

Historically, unfractionated heparin and low-molecular-weight heparins have been recognized as the cornerstones of treatment for DVT (179) and MI (180). Due to the inflammatory nature of thrombotic diseases, it is intriguing that heparin has anti-inflammatory effects (181, 182). Heparins can not only hinder NETs formation as shown by treatment of *in vitro* human neutrophils and *in vivo* healthy volunteers (183), but can also target existing NETs *in vitro* in various ways. In NETs, two major enzymes, neutrophil elastase and cathepsin G, were shown to be inhibited by heparin (184, 185). By binding and disassembling histones, heparin inhibits NETs’ pro-thrombotic properties *in vitro* (11) and *in vivo* in mice (102, 186). Heparin’s antithrombotic properties in human plasma have been counteracted by excessive release of histones during cell death and NETosis, according to an *in vitro* study (187). Sevuparin, a low-anticoagulant heparin analog, inhibits NE and histone H4 proteins associated with NETs secreted by human neutrophils *in vitro* (188). Another study showed that non-anticoagulant heparin prevented histone-mediated cytotoxicity in a murine sepsis model and improved survival (189). There are many types of heparin, which are commonly used in clinics. Thus, an appropriate dose of heparin under close monitoring might be an important adjunct therapy to reduce NET burdens without increasing bleeding complications. Despite this, there are few reports on heparin-targeted treatment of ET-related diseases, and further research is needed.

### Acetylsalicylic acid (aspirin), a non-steroidal drug with an antithrombotic effect

Aspirin, an antiplatelet drug, is used for prevention of arterial thromboses for many years. Previous studies reported that platelets could activate NETosis via the platelet-neutrophil interaction and facilitate innate immunity (51, 88). During endotoxemia and in septic conditions, the binding of glycoprotein 1bα (GP1bα) on platelets and αMβ2 (MAC-1) on neutrophils could activate NETosis and promote the release of NETs *in vitro* (190, 191). Otherwise, platelet activation by TLR2 and TLR4 results in the release of Pselectin, which binds to the neutrophil receptor (PSGL-1), leading to the development of NETosis as demonstrated in mice (59, 192, 193). When exposed to LPS, platelets could also be activated by arachidonic acid and thrombin, and the formation of NETs increased (51, 194). As platelet and neutrophil interactions mediate NETosis, inhibiting these interactions with antiplatelet therapy can prevent NETs formation (195, 196). The activation of platelet could be inhibited by aspirin had been confirmed in primary graft dysfunction murine models (197). Aspirin pretreatment of mice with endotoxin-triggered acute lung injury decreased the formation of NETs and the severity of lung injury (194, 196). In a vitro study of neutrophils, Lapponi et al. demonstrated that NETs formation was inhibited after the intervention with aspirin (198). Based on these results, aspirin may be useful in treating pathologic NETosis induced by platelets. However, aspirin predisposes patients to stomach ulcers, so more studies are needed to determine what conditions could benefit from taking aspirin without experiencing too many side effects.

### Other substances potential inhibit NETosis

In addition to the above-mentioned widely used drugs, there were also other substances reported recently in a few studies that might inhibit NETosis, such as cyclosporine A (199), prostaglandins E2 (200, 201), recombinant human thrombomodulin (202), activated protein C (203), metformin (204), and Vitamin D (205). Although uncertainties still remain in these findings, they represented different directions for future studies.

## Conclusions and perspectives

**FIGURE 2:**
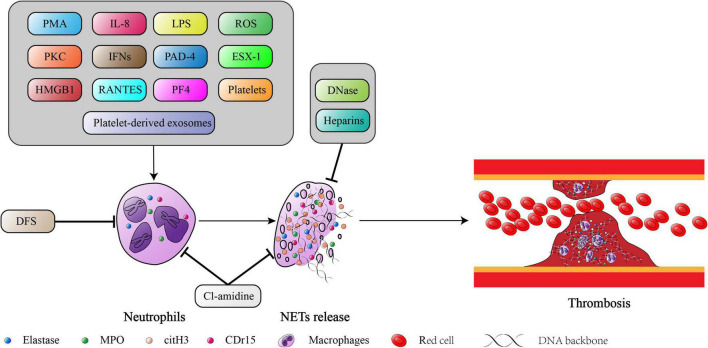
The summarized mechanism of NETs formation and its consequences in thrombosis. The figure was constructed by Adobe Illustrator.

Although the formation and aggregation of ETs actively limits the spread of inflammatory mediators and pathogens, excessive accumulation of ETs is also a cause of thrombotic diseases. A summary of these studies highlighted and discussed above, ETs clearly play an important role in the initiation and progression of thrombosis (Figure 2). Based on these findings, ETs have been considered new potential therapeutic targets. Until now, the digestion of established ETs and prevention of new ETs formation to alleviate the severity of thrombosis remains controversial. Although *in vitro* and *in vivo* data are promising, more in-depth studies are needed to evaluate the clinical safety and benefits of anti-ETotic regimens.

## Author contributions

TH, XT, and DG conceived the review. TH, HT, CL, YS, and DY wrote the manuscript and designed the figures. All authors contributed to the article and approved the submitted version.

## Abbreviations

AAA: Abdominal aortic aneurysm
aggETs: aggregated ETs
citH3: Citrullinated histone H3
COVID-19: Coronavirus disease 19
DFS: Deferasirox
DNase: Deoxyribonuclease
dsDNA: Double-stranded DNA
DVT: Deep vein thrombosis
ET: Extracellular traps
HMGB1: High mobility group box 1
ICA: Internal carotid artery
IFNs: Type I interferons
IL: Interleukin
LPS: Lipopolysaccharide
MCA: Middle cerebral artery
MI: Myocardial infarction
MPO: Myeloperoxidase
NE: Neutrophil elastase
NETs: Neutrophil extracellular traps
METs: Macrophage extracellular traps
MCETs: Mast cell extracellular traps
PAD: Peripheral artery disease
PAD-4: Peptidylarginine deiminase 4
PE: Pulmonary embolism
PF4: Platelet factor 4
PKC: Protein kinase C
PMA: Phorbol myristate acetate
RANTES: Regulated on activation normal t-cell expressed and secrected
ROS: Reactive oxygen species
VTE: Venous thromboembolism
vWF: von Willebrand factor.
